# Supplementary figures and images for: Integrative proteomic and phosphoproteomic profiling of prostate cell lines
Source: PLoS One. 2019 Nov 1;14(11):e0224148. doi: 10.1371/journal.pone.0224148 (PMC6824562; doi:10.1371/journal.pone.0224148)

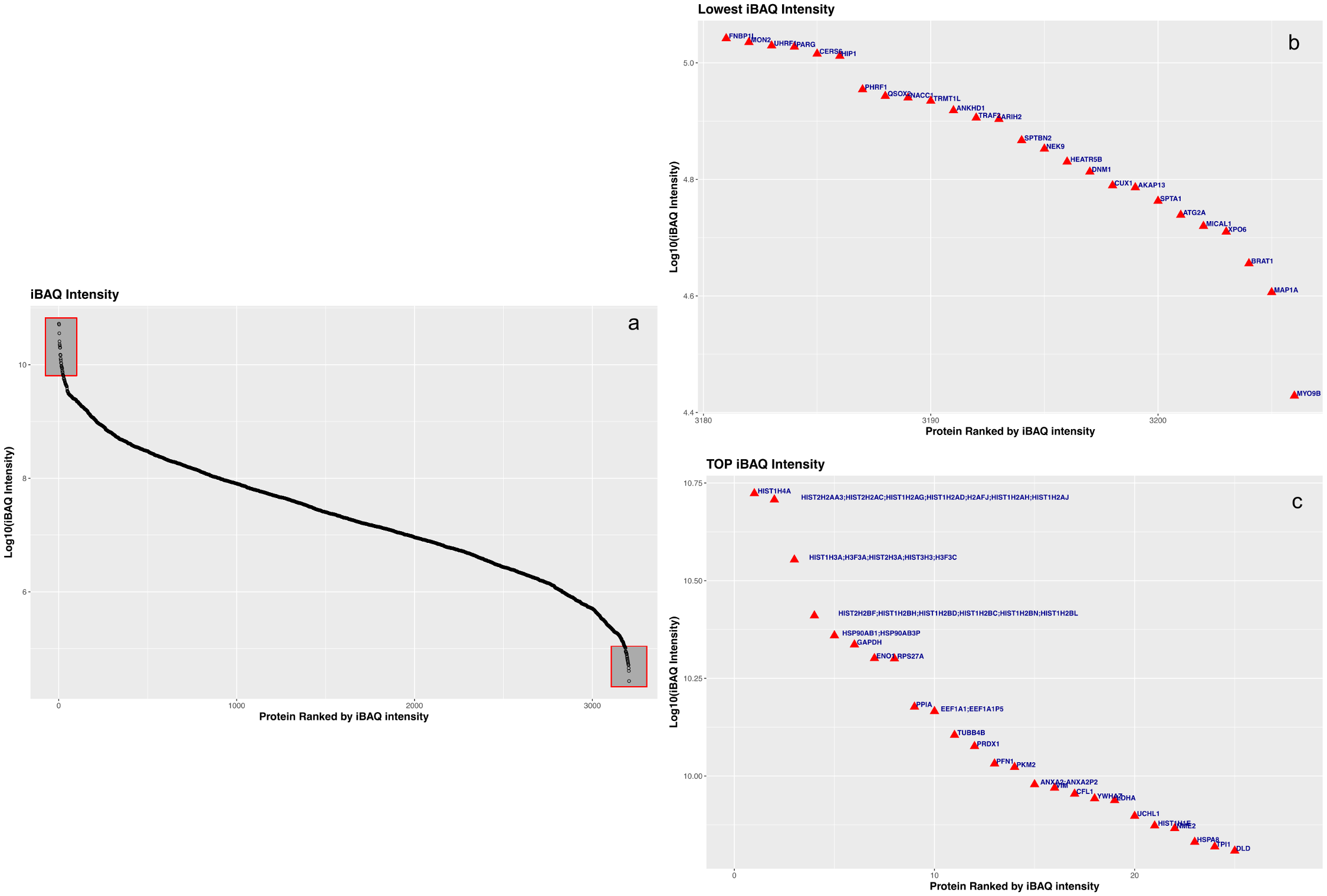

Supplement: S1 Fig — (a) Ranking of the absolute abundance using the IBAQ intensity. The expression values of every protein in the three replicates of the four studied cell lines were considered. (b) Zoom on the left box in (a) displaying the 25 less abundant proteins. (c) Zoom on the right box in (a) displaying the 25 most abundant proteins. (TIF) [file pone.0224148.s001.tif]

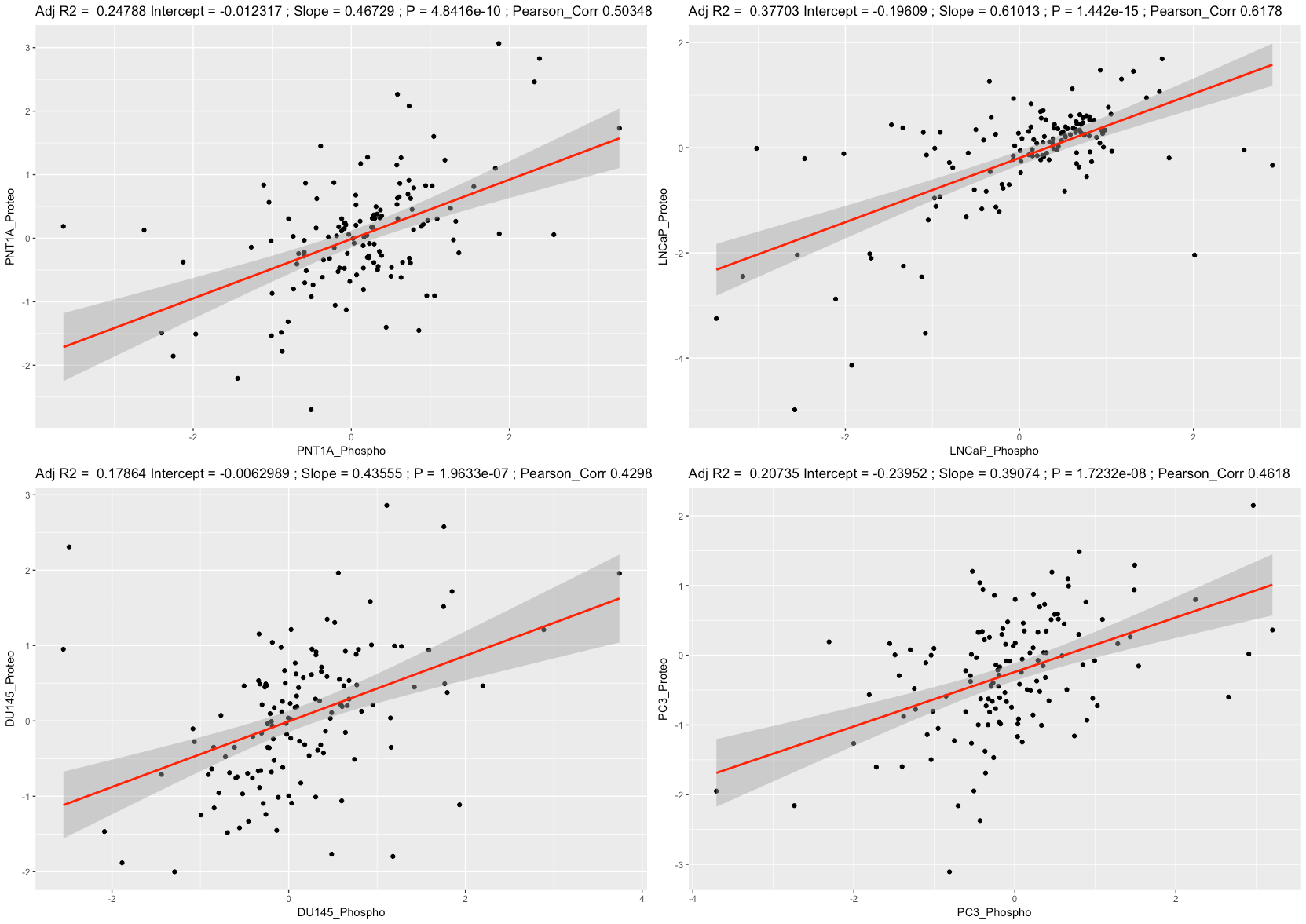

Supplement: S2 Fig — We computed for each cell line the correlation between the expression values of the 135 proteins that were quantified both at the proteomic and the phosphoproteomic levels. For proteomics data, we computed the mean of the three replicated. For phosphoproteomics data, we computed the mean for all the phosphosites belonging to the same protein. (TIF) [file pone.0224148.s002.tif]

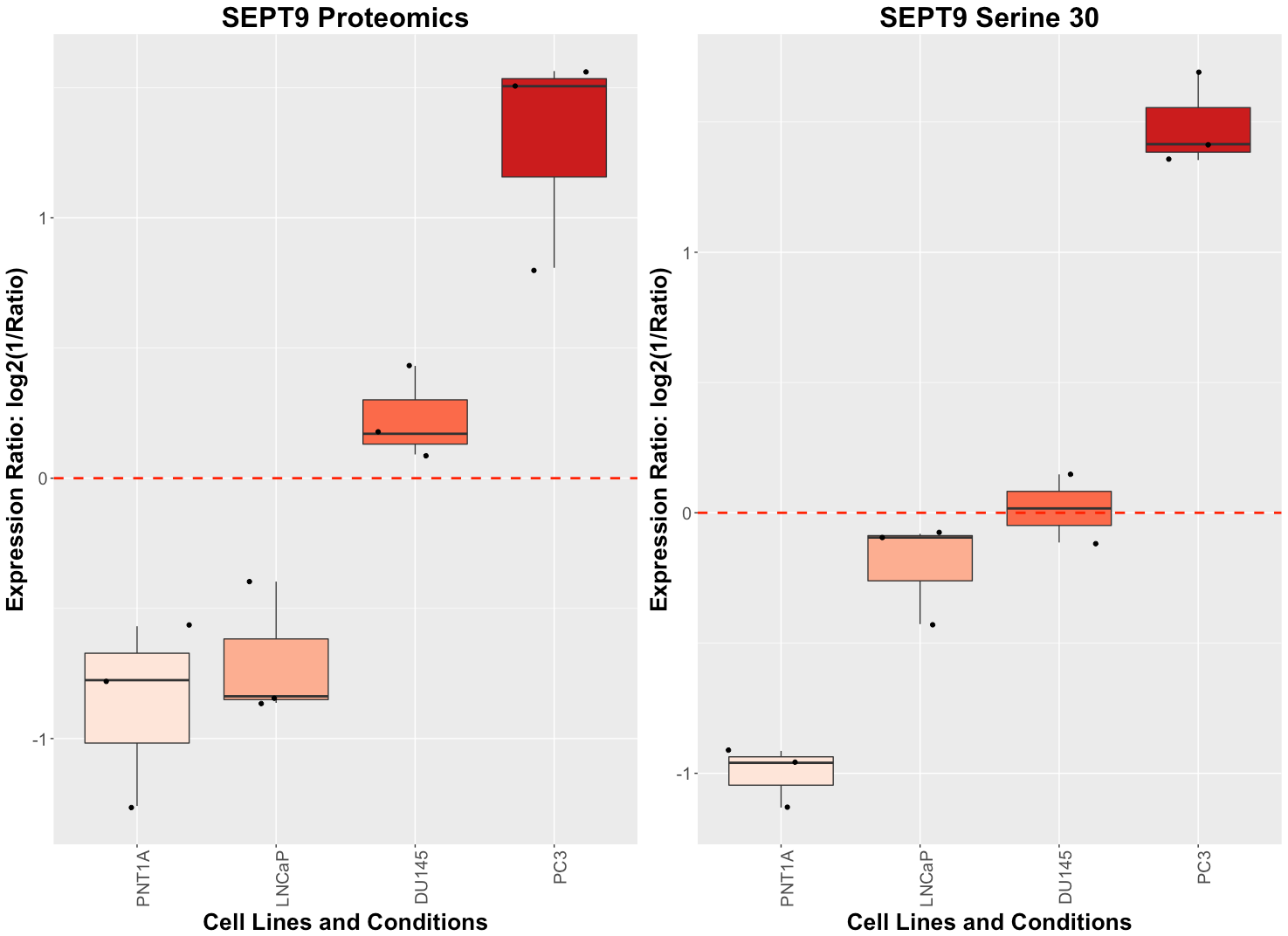

Supplement: S3 Fig — (a) Boxplot showing the SEPT9 protein expression values in the four cell lines under study. (b) Boxplot revealing the SEPT9 Serine-30 phosphosite expression values in the four cell lines under study. (TIF) [file pone.0224148.s003.tif]

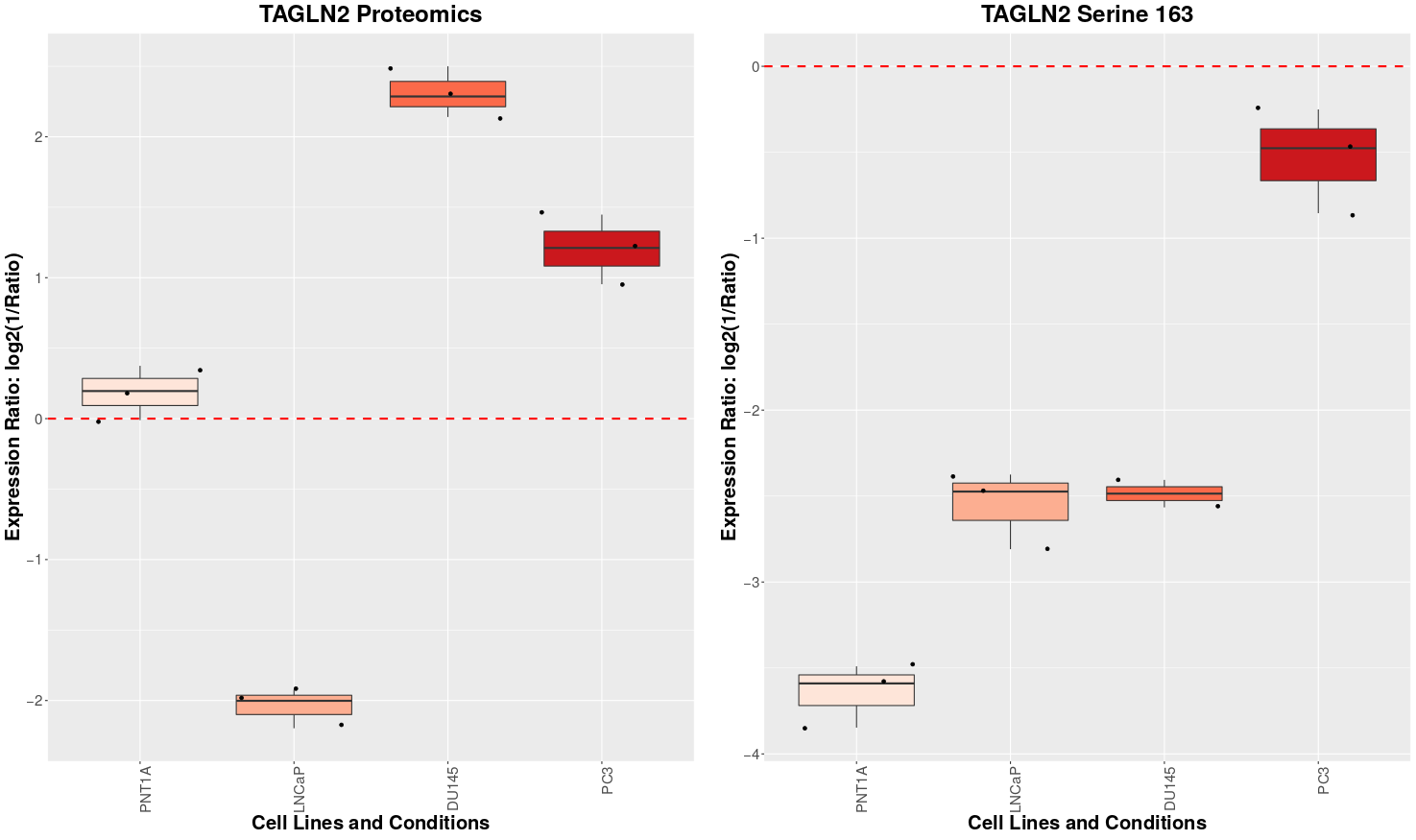

Supplement: S4 Fig — (a) Boxplot showing the TAGLN2 protein expression values in the four cell lines under study. (b) Boxplot revealing the TAGLN2 Serine-163 phosphosite expression values in the four cell lines under study. (TIFF) [file pone.0224148.s004.tiff]

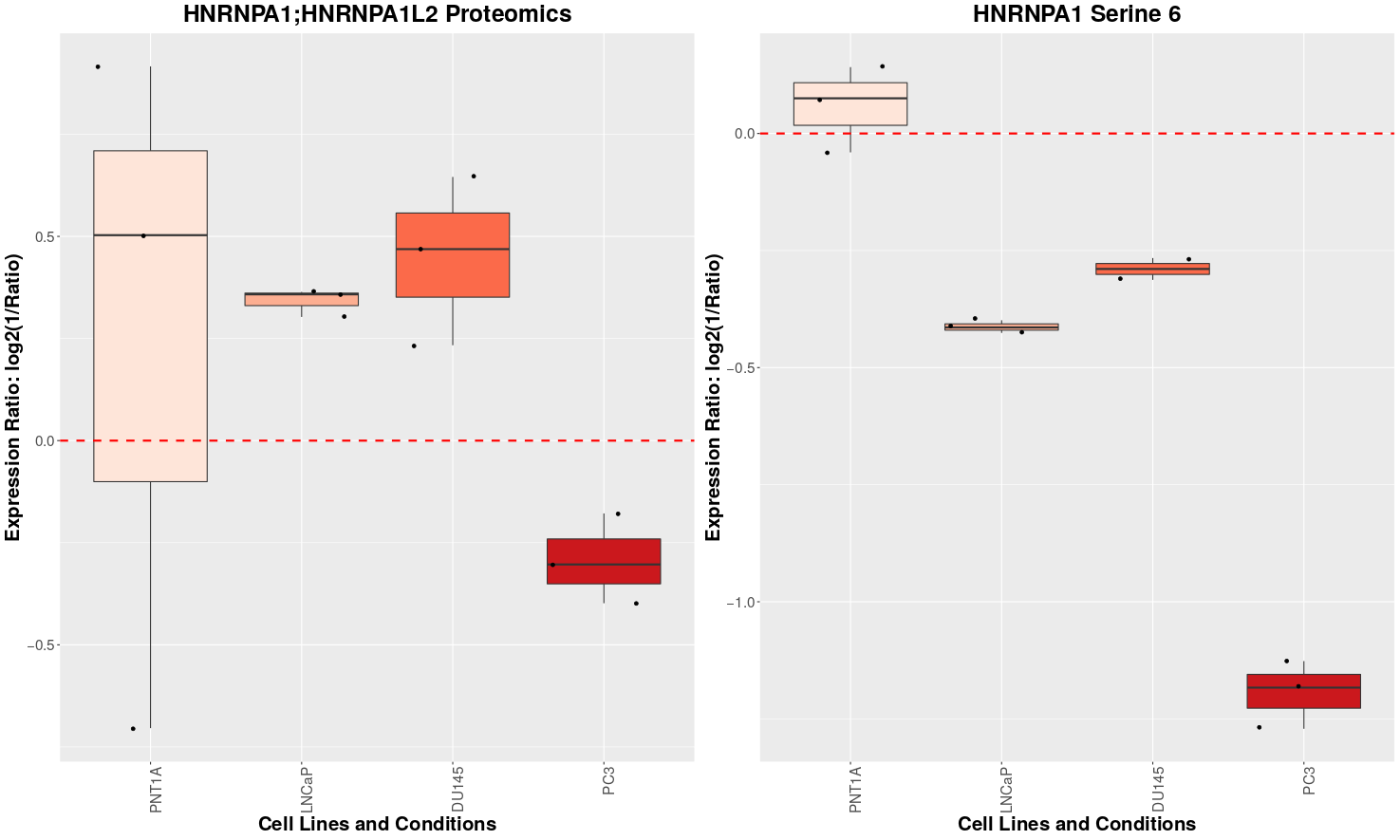

Supplement: S5 Fig — (a) Boxplot showing the HNRNPA1 protein expression values in the four cell lines under study. (b) Boxplot revealing the HNRNPA1 Serine-6 phosphosite expression values in the four cell lines under study. (TIFF) [file pone.0224148.s005.tiff]
