# Supplementary material for: Integrative proteomic and phosphoproteomic profiling of prostate cell lines
Source: PLoS One. 2019 Nov 1;14(11):e0224148. doi: 10.1371/journal.pone.0224148 (PMC6824562; doi:10.1371/journal.pone.0224148)
Supplement: Attachment — Submitted filename: Response To Reviewers.pdf [file pone.0224148.s011.pdf]

PONE-D-19-20799
Integrative proteomic and phosphoproteomic profiling of prostate cell lines
PLOS ONE

Tuesday, August 13, 2019 08:08

Dear Dr Valdeolivas

Thank you for submitting your manuscript to PLOS ONE. After careful consideration, we feel that it has merit but does not fully meet PLOS ONE's publication criteria as it currently stands. Therefore, we invite you to submit a revised version of the manuscript that addresses the points raised during the review process.
A significant number of concerns have been raised by 3 expert Reviewers; the comments are technical, scientific and do concern also the text.
We would appreciate receiving your revised manuscript that addresses all comments.
When you are ready to submit your revision, log on to https://www.editorialmanager.com/pone/ and select the 'Submissions Needing Revision' folder to locate your manuscript file.
If you would like to make changes to your financial disclosure, please include your updated statement in your cover letter.

To enhance the reproducibility of your results, we recommend that if applicable you deposit your laboratory protocols in protocols.io, where a protocol can be assigned its own identifier (DOI) such that it can be cited independently in the future. For instructions see:
http://journals.plos.org/plosone/s/submission-guidelines#loc-laboratory-protocols

Please include the following items when submitting your revised manuscript:

- A rebuttal letter that responds to each point raised by the academic editor and reviewer(s). This letter should be uploaded as separate file and labeled 'Response to Reviewers'.
- A marked-up copy of your manuscript that highlights changes made to the original version. This file should be uploaded as separate file and labeled 'Revised Manuscript with Track Changes'.
- An unmarked version of your revised paper without tracked changes. This file should be uploaded as separate file and labeled 'Manuscript'.

Please note while forming your response, if your article is accepted, you may have the opportunity to make the peer review history publicly available. The record will include editor decision letters (with reviews) and your responses to reviewer comments. If eligible, we will contact you to opt in or out.
We look forward to receiving your revised manuscript.

Kind regards,

Lucia R. Languino, Ph.D.
Academic Editor
PLOS ONE

Dear Dr Lucia Languino

Many thanks for your feedback regarding our paper entitled “Integrative proteomic and phosphoproteomic profiling of prostate cell lines”. We now have studied reviewer’s comments in detail, and provide point-by-point responses, together with a revised version of our manuscript which includes two new supplementary figures. We also modified the labels, axis and titles of some of the manuscript figures to improve their clarity.

We considered publishing our protocol, but we think that it might not be appropriate in this case. Indeed, as referred in the methods section, we mainly followed the spike-in SILAC protocol published by Mathias Mann and his team (Geiger et al. 2010, Geiger et al. 2011).

Finally, we updated our financial disclosure according to your recommendations as follows: *“This work was supported by the French Plan Cancer 2009-2013 (Systems Biology call). The funders had no role in study design, data collection and analysis, decision to publish, or preparation of the manuscript. The funder ProGeLife provided support in the form of salaries for author AV, but had no role in the study design, data collection and analysis, decision to publish, or preparation of the manuscript. This commercial affiliation does not alter our adherence to PLOS ONE policies on sharing data and materials. The specific roles of the authors are articulated in the ‘author contributions’ section”*. We also included this paragraph in an updated version of the cover letter.

Geiger T, Cox J, Ostasiewicz P, Wisniewski JR, Mann M. Super-SILAC mix for quantitative proteomics of human tumor tissue. *Nat Methods.* 2010;7(5):383-385.
Geiger T, Wisniewski JR, Cox J, et al. Use of stable isotope labeling by amino acids in cell culture as a spike-in standard in quantitative proteomics. *Nat Protoc.* 2011;6(2):147-157.

On the behalf of the authors,

Journal requirements:

When submitting your revision, we need you to address these additional requirements.

1. Please ensure that your manuscript meets PLOS ONE's style requirements, including those for file naming. The PLOS ONE style templates can be found at http://www.journals.plos.org/plosone/s/file?id=wjVg/PLOSOne_formatting_sample_main_body.pdf and http://www.journals.plos.org/plosone/s/file?id=ba62/PLOSOne_formatting_sample_title_authors_affiliations.pdf

We have now revised our manuscript and file naming aiming at meeting the PLOS ONE's style requirements. We put special emphasis in the correction of author's affiliations. We however must follow the requirements from our institutions which have very strict policies concerning this point. We hope to find a solution on this point.

2. We note that you have included the phrase "data not shown" in your manuscript. Unfortunately, this does not meet our data sharing requirements. PLOS does not permit references to inaccessible data. We require that authors provide all relevant data within the paper, Supporting Information files, or in an acceptable, public repository. Please add a citation to support this phrase or upload the data that corresponds with these findings to a stable repository (such as Figshare or Dryad) and provide and URLs, DOIs, or accession numbers that may be used to access these data. Or, if the data are not a core part of the research being presented in your study, we ask that you remove the phrase that refers to these data.

Ok. we decided to remove it as this was not a core part of the study.

3. Thank you for stating the following in the Financial Disclosure section:
"This work was supported by the French "Plan Cancer 2009-2013" (Systems Biology call). The funders had no role in study design, data collection and analysis, decision to publish, or preparation of the manuscript."

We added the sentence: "This work was supported by the French "Plan Cancer 2009-2013" (Systems Biology call). The funders had no role in study design, data

collection and analysis, decision to publish, or preparation of the manuscript” to the funding statement and to the cover letter.

We note that one or more of the authors are employed by a commercial company: 'ProGeLife, Marseille, France'.
a) Please provide an amended Funding Statement declaring this commercial affiliation, as well as a statement regarding the Role of Funders in your study. If the funding organization did not play a role in the study design, data collection and analysis, decision to publish, or preparation of the manuscript and only provided financial support in the form of authors' salaries and/or research materials, please review your statements relating to the author contributions, and ensure you have specifically and accurately indicated the role(s) that these authors had in your study. You can update author roles in the Author Contributions section of the online submission form.
Please also include the following statement within your amended Funding Statement.
“The funder provided support in the form of salaries for authors [insert relevant initials], but did not have any additional role in the study design, data collection and analysis, decision to publish, or preparation of the manuscript. The specific roles of these authors are articulated in the ‘author contributions’ section.”

We added the sentence: “The funder ProGeLife provided support in the form of salaries for author AV, but had no role in the study design, data collection and analysis, decision to publish, or preparation of the manuscript. The specific roles of the authors are articulated in the ‘author contributions’ section” to the funding statement and to the cover letter.

If your commercial affiliation did play a role in your study, please state and explain this role within your updated Funding Statement.

b) Please also provide an updated Competing Interests Statement declaring this commercial affiliation along with any other relevant declarations relating to employment, consultancy, patents, products in development, or marketed products, etc.
Within your Competing Interests Statement, please confirm that this commercial affiliation does not alter your adherence to all PLOS ONE policies on sharing data and materials by including the following statement: "This does not alter our adherence to PLOS ONE policies on sharing data and materials.” (as detailed online in our guide for authors http://journals.plos.org/plosone/s/competing-interests) . If this adherence statement is not accurate and there are restrictions on sharing of data and/or materials,

please state these. Please note that we cannot proceed with consideration of your article until this information has been declared.
Please include both an updated Funding Statement and Competing Interests Statement in your cover letter. We will change the online submission form on your behalf.
Please know it is PLOS ONE policy for corresponding authors to declare, on behalf of all authors, all potential competing interests for the purposes of transparency. PLOS defines a competing interest as anything that interferes with, or could reasonably be perceived as interfering with, the full and objective presentation, peer review, editorial decision-making, or publication of research or non-research articles submitted to one of the journals. Competing interests can be financial or non-financial, professional, or personal. Competing interests can arise in relationship to an organization or another person. Please follow this link to our website for more details on competing interests: http://journals.plos.org/plosone/s/competing-interests

We added the sentence “This commercial affiliation does not alter our adherence to PLOS ONE policies on sharing data and materials.” to the funding statement and to the cover letter. Please do not hesitate to contact us if we did not understand something correctly.

Reviewers' comments:

Reviewer's Responses to Questions

Comments to the Author

1. Is the manuscript technically sound, and do the data support the conclusions?

The manuscript must describe a technically sound piece of scientific research with data that supports the conclusions. Experiments must have been conducted rigorously, with appropriate controls, replication, and sample sizes. The conclusions must be drawn appropriately based on the data presented.

Reviewer #1: No

Reviewer #2: Yes

Reviewer #3: Partly

---

2. Has the statistical analysis been performed appropriately and rigorously?

Reviewer #1: Yes

Reviewer #2: Yes

Reviewer #3: Yes

---

3. Have the authors made all data underlying the findings in their manuscript fully available?

The PLOS Data policy requires authors to make all data underlying the findings described in their manuscript fully available without restriction, with rare exception (please refer to the Data Availability Statement in the manuscript PDF file). The data should be provided as part of the manuscript or its supporting information, or deposited to a public repository. For example, in addition to summary statistics, the data points behind means, medians and variance measures should be available. If there are restrictions on publicly sharing data—e.g. participant privacy or use of data from a third party—those must be specified.

Reviewer #1: Yes

Reviewer #2: Yes

Reviewer #3: Yes

---

4. Is the manuscript presented in an intelligible fashion and written in standard English?

PLOS ONE does not copyedit accepted manuscripts, so the language in submitted articles must be clear, correct, and unambiguous. Any typographical or grammatical errors should be corrected at revision, so please note any specific errors here.

Reviewer #1: Yes

Reviewer #2: Yes

Reviewer #3: Yes

5. Review Comments to the Author

Please use the space provided to explain your answers to the questions above. You may also include additional comments for the author, including concerns about dual publication, research ethics, or publication ethics. (Please upload your review as an attachment if it exceeds 20,000 characters)

Reviewer #1:

There are several studies in the literature about prostate cancer proteomics. However, in some cases these previous publications yielded data with limited reproducibility. It is sometimes difficult to distinguish between data which have implications in the clinic and others. Although data quality is acceptable, there are some questions about the conclusions.

We agree that reproducibility is a major issue in cancer research, and that the translation from observations to clinics is often uncertain. Here are few choices we made to try to mitigate these issues:

- We avoided the imputation of missing values in our proteomics datasets, and performed stringent filtering to reduce the impact of technical noise;
- We tried to be particularly cautious in the wording of the manuscript, and to not overstate the clinical implications of the candidates obtained during the exploration of our datasets;
- We selected cell lines widely used as models in prostate cancer research;
- We carefully checked the literature to evaluate if we retrieve (and thereby reproduce) proteins already described associated with prostate cancer.

Specific points:

1. the reason for comparison of sensitive and resistant cell lines are not clear. This could imply that androgen receptor expression decreases during tumor progression what is not the case. It is difficult to draw any conclusion from this comparison.

We are not sure to understand this comment. Indeed, the two castration-resistant cell lines do lack the androgen receptor, and the castration-sensitive cell line expresses it (Pienta et al. 2008, Cunningham et al. 2015). The goal of the comparison of sensitive versus resistant cells was thereby to assess pathways that could be differentially activated in these cell lines, and thereby point towards mechanisms implemented by the resistant cell lines to overcome, among others, the absence of the androgen receptor. Several mechanisms are known to be involved in the emergence of the malignant progression of prostate cancer and resistance phenotype, through AR-related and non-AR-related pathways. For example, post-transcriptional modification using miRNA, epigenetic alterations, alternative splicing or gene fusion are parts of the hallmark of CRPC (Katsogiannou M., Cancer Treat Rev. 2015). We identified several proteins involved in these mechanisms in our proteomics screening. In 30% of CRPC, the AR promoter region has been described to be hypermethylated, resulting in the loss of AR expression in those tumors (Suzuki H, Endocr Relat Cancer 2003).

In the manuscript, we clarified the introduction to state precisely that resistance is associated with androgen receptor pathway changes (line 8 in the introduction). "This progression involves several molecular mechanisms such as ligand-independent androgen receptor activation, androgen receptor (AR) loss or adaptive upregulation of anti-apoptotic genes (for review [3])".

Pienta KJ, Abate-Shen C, Agus DB, Attar RM, Chung LW, Greenberg NM, Hahn WC, Isaacs JT, Navone NM, Peehl DM, Simons JW, Solit DB, Soule HR, VanDyke TA, Weber MJ, Wu L, Vessella RL. The current state of preclinical **prostate** cancer animal models. **Prostate**. **2008**May 1;68(6):629-39. doi: 10.1002/pros.20726

CunninghamD, You Z. In vitro and in vivo model systems used in **prostate** cancer research._J Biol Methods. **2015**;2(1). pii: e17.

2. the descriütion of human cell lines in results is not necessary. They are very well known in scientific community.

These prostate cell lines are indeed well-known in the prostate cancer research community. We however expect the dataset we are producing to be used as a resource by other researchers with diverse expertise, such as bioinformatics, and think it's important to state their biological features in details. We however removed the description of the cell lines from the Material and Methods sections to reduce redundancies.

3. In the Results section, there are too many repetitions of that what has already been presented in Materials and Methods.

We have reviewed the Results and Material & Methods sections in details and removed redundancies.

4. it is not clear whether this manuscript could confirm previous findings in the proteomic field.

Apart from the proteins for which we manually curated the literature to validate their previous implication in prostate cancer, and that are described in the text, we also considered comparing our results with existing "omics" large-scale datasets. We identified prostate cancer publications focusing on tissue proteomics (Iglesias-Gato et al. 2016) and on cell line & tissue phosphoproteomics (Drake et al. 2016). We also found an update of Iglesias-Gato et al. 2016 on the proteome of prostate cancer bone metastasis (Iglesias-Gato et al. 2018). Finally, genomics data are also available for ~60 cancer cell lines in the NCI60 resource. We discuss below the comparison of our dataset with each of these resources. Please note that this answer is similar to the one proposed for Reviewer 2 first comment.

Iglesias-Gato, D. et al. The Proteome of Primary Prostate Cancer. European Urology 69, 942–952 (2016).

Drake, J. M. et al. Phosphoproteome Integration Reveals Patient-Specific Networks in Prostate Cancer. Cell 166, 1041–1054 (2016).

Iglesias-Gato, D. et al. The Proteome of Prostate Cancer Bone Metastasis Reveals Heterogeneity with Prognostic Implications. Clin. Cancer Res. 24, 5433–5444 (2018).

- Comparison with NCI60 genomics data resource

We checked the NCI-60 tumor cell line screen (https://dtp.cancer.gov/discovery_development/nci-60/), which provide genomics data for a large

panel of reference cell lines. Our goal was to try to link genomics mutations in cell lines with proteomics level deregulations. However, only 2 of the 4 cell lines that we profiled in our experiment are described in this database (DU145 and PC3), and we decided not to pursue in this direction.

- Drake et al. (2016)

In Drake et al., the authors integrate transcriptomics and phosphoproteomics (but no proteomics) data for many tumor tissues and 3 prostate cancer cell lines. We studied their paper in-depth, in particular the Figure 1, which shows that the cell lines data were clustering apart from the tissue data. We contacted the authors when the paper was published, and they gave us a more detailed presentation of their Figure 1. Unfortunately, we have only the DU145 cell line in common, and we were thereby not able to compare our clusterings. In addition, a direct comparison of our phosphoproteomics raw data is not pertinent as, contrarily to us, their protocole implies vanadate (a protein-phosphotyrosine phosphatase inhibitor) treatment, in order to increase the detection of the phospho-tyrosine.

- Iglesia-Gato et al. 2016

We also compared the proteomics data we have generated with the ones produced in Iglesias-Gato 2016. We selected the normalised ratios (before imputation of missing values) of the 9829 identified proteins in the Iglesias-Gato dataset. In our dataset, we took the normalised ratios of the 3219 identified proteins. The heatmap below was created based on the Pearson correlation values between the expression levels of 2673 proteins identified in both experiments. We can observe that cell lines clusterize apart from tissue data. This clustering is similar to the one obtained by in prostate cancer transcriptomics and phosphoproteomics (Drake et al. 2016) as well as for other cancer types and omics data (Domcke et al. 2013). It has also been observed for breast cancer that among different types of omics, proteins levels had the lowest correlation between tumors and cell lines (Jiang et al. 2016). Overall, these observations highlight the large dissimilarities between tissue samples and cell lines, emphasizing the need to obtain molecular profiles from both types of resources.

Domcke, S., Sinha, R., Levine, D. A., Sander, C. & Schultz, N. Evaluating cell lines as tumour models by comparison of genomic profiles. Nature Communications 4, 2126 (2013).

Drake, J. M. et al. Phosphoproteome Integration Reveals Patient-Specific Networks in Prostate Cancer. Cell 166, 1041–1054 (2016).

Jiang G, Zhang S, Yazdanparast A, et al. Comprehensive comparison of molecular portraits between cell lines and tumors in breast cancer. BMC Genomics. 2016;17 Suppl 7(Suppl 7):525.

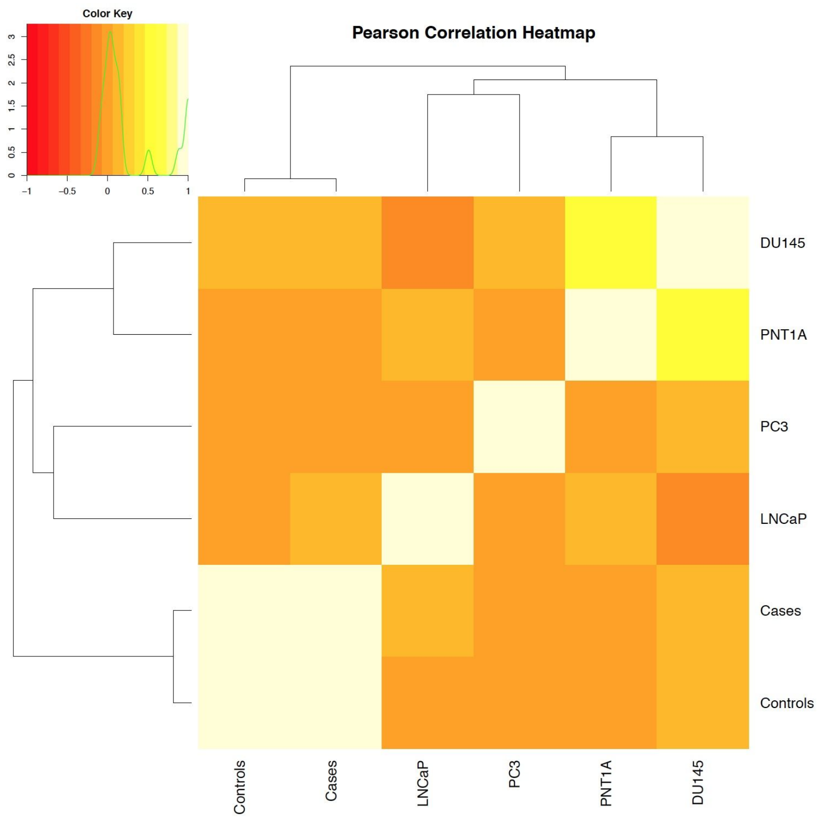

We also conducted a meta-analysis using the R package “MetaDE”. Our goal was to compare the protein level trends among tissue and cell lines. We used a Random Effects Model (REM) approach for the meta-analysis, since it allows heterogeneity in the effect sizes between the different datasets (Tissue VS cell lines). We took PNT1A as the reference for our SILAC experiment. Proteins were selected as those displaying an FDR corrected p-value < 0.05. The results are the following:

- **LNCaP**: 114 proteins have a similar behaviour in cell lines as compared to tumor tissues. This means that the expression level changes of these proteins between LNCaP and PNT1A are similar to those between control tissue and tumorigenic tissue.
- **DU145**: 81 proteins have a similar behaviour.
- **PC3**: 73 proteins have a similar behaviour.

These results indicate that LNCaP is the cell line with protein levels displaying the most similar behavior as compared to the tumor tissue.

- Iglesia-gato 2018

We finally applied our previously mentioned comparison protocol to the data produced by Iglesias-Gato in 2018. In this case, they identify 9828 proteins, 2673 of them also match with our

identified proteins. Here, we also observe that our protein expression data clusterize separately from the one resulting from tissue samples.

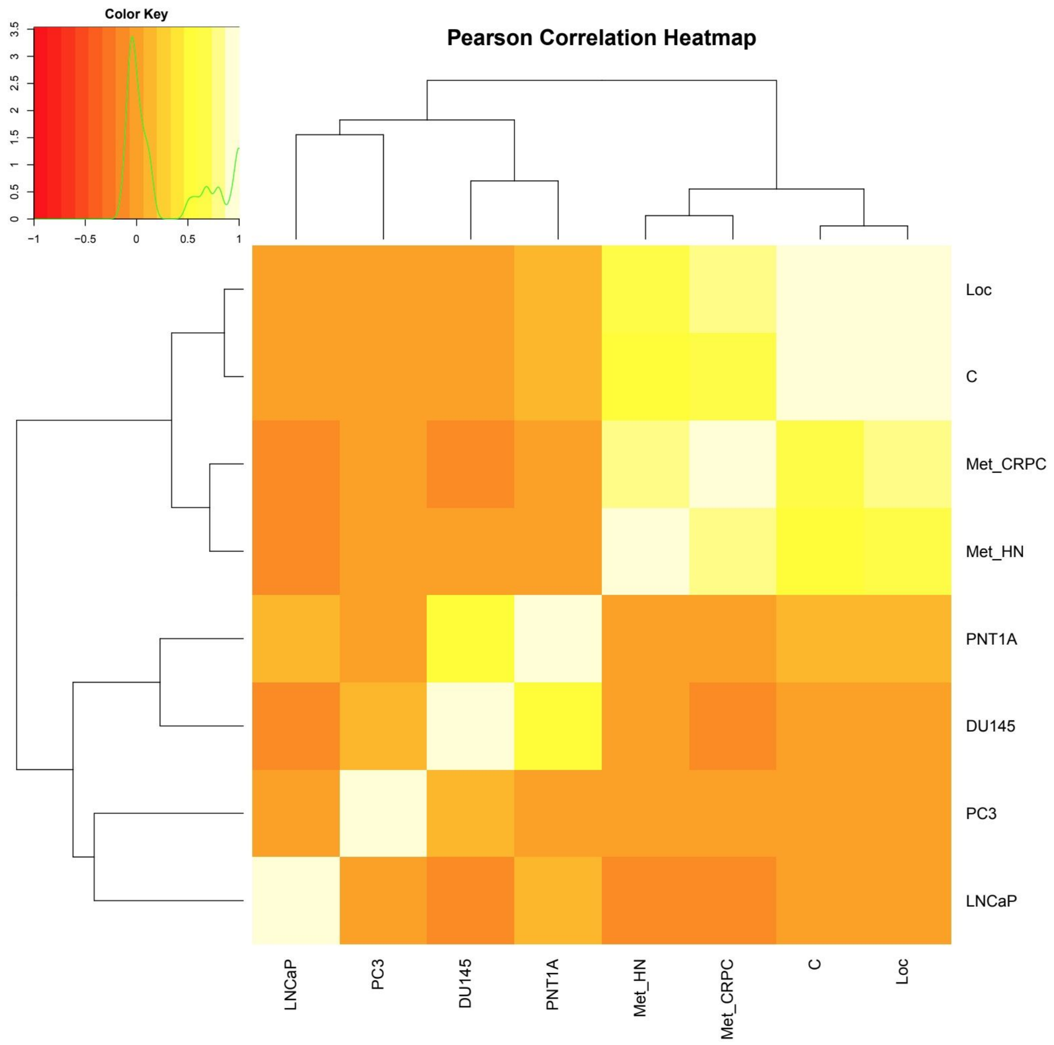

C: benign neighboring prostate tissue
Loc: gland localized prostate tumors (Primary tumors)
Met-HN: Bone metastasis Hormone-naive.
Met-CRPC: Bone metastasis Castration resistant prostate cancer

Overall, the main strengths of our work are to profile four widely used prostate cell lines and to do this both at the proteomics and phosphoproteomics levels. In addition, as shown in the previous comparisons, our datasets are complementary to the recent literature on prostate cancer transcriptomics, proteomics or phosphoproteomics, and could be useful for the community working with these cell lines in routine. Furthermore, we also propose a bioinformatics exploration of our datasets, as well as their integration into a molecular network,

allowing the identification of processes that are not detectable with a traditional analyses of differentially expressed proteins/sites.

Reviewer #2:

M Katsogiannou et al presented a study that aimed to investigate and explore the proteome and phosho-proteome of four well established prostate cancer cell lines. Utilizing a SILAC-based Mass Spectrometry approach, the data show set of proteins that are commonly and highly expressed in all four cell lines, as well as differentially expressed proteins between castrate-resistant and castrate-sensitive cells. Other comparisons such as proteins up-regulated in cancer cell lines compared to non-tumorigenic cell is demonstrated. Phospho-proteomic data is also presented for the cell lines including presentation of the proteome and phosphor-proteome in a molecular network to identify candidate biomarkers for Prostate cancer.

We thank the reviewer for this accurate and detailed summary of our manuscript.

Overall, the study is well-done and provides a starting point to further explore the role of potential candidate proteins identified through this study in prostate cancer. Although the study adds some new potential proteins to the list as target molecules and biomarkers, it reconfirms a lot of information that is already published in the field.

Many thanks. Indeed, one of the manuscript's main goals is to provide a resource as a starting point for further exploration of proteins related to PC progression.

1)There are other studies that have used proteomic -based approaches on prostate cancer cells or conditioned media from prostate cancer cells? What is the novelty of the approach used in this study?

Apart from the proteins for which we manually curated the literature to validate their previous implication in prostate cancer, and that are described in the text, we also considered comparing our results with existing “omics” large-scale datasets. Importantly, we did not identify other proteomics/phosphoproteomics approaches focusing on prostate cell lines, but we identified prostate cancer publications focusing on tissue proteomics (Iglesias-Gato et al. 2016) and on cell line & tissue phosphoproteomics (Drake et al. 2016). We also found an update of Iglesias-Gato et al. 2016 on the proteome of prostate cancer bone metastasis (Iglesias-Gato et al. 2018). Finally, genomics data are also available for ~60 cancer cell lines in the NCI60

resource. We discuss below the comparison of our dataset with each of these resources. Please note that this answer is similar to the one proposed for Reviewer 1 fourth comment.

Iglesias-Gato, D. et al. The Proteome of Primary Prostate Cancer. European Urology 69, 942–952 (2016).

Drake, J. M. et al. Phosphoproteome Integration Reveals Patient-Specific Networks in Prostate Cancer. Cell 166, 1041–1054 (2016).

Iglesias-Gato, D. et al. The Proteome of Prostate Cancer Bone Metastasis Reveals Heterogeneity with Prognostic Implications. Clin. Cancer Res. 24, 5433–5444 (2018).

- Comparison with NCI60 genomics data resource

We checked the NCI-60 tumor cell line screen (https://dtp.cancer.gov/discovery_development/nci-60/), which provide genomics data for a large panel of reference cell lines. Our goal was to try linking genomics mutations in cell lines with proteomics level deregulations. However, only 2 of the 4 cell lines that we profiled in our experiment are described in this database (DU145 and PC3), and we decided not to pursue in this direction.

- Drake et al. (2016)

In Drake et al., the authors integrate transcriptomics and phosphoproteomics (but no proteomics) data for many tumor tissues and 3 prostate cancer cell lines. We studied their paper in-depth, in particular the Figure 1, which shows that the cell lines data were clustering apart from the tissue data. We contacted the authors when the paper was published, and they gave us a more detailed presentation of their Figure 1. Unfortunately, we have only the DU145 cell line in common, and we were thereby not able to compare our clusterings. In addition, a direct comparison of our phosphoproteomics raw data is not pertinent as, contrarily to us, their protocole implies vanadate (a protein-phosphotyrosine phosphatase inhibitor) treatment, in order to increase the detection of the phospho-tyrosine.

- Iglesia-Gato et al. 2016

We also compared the proteomics data we have generated with the ones produced in Iglesias-Gato 2016. We selected the normalised ratios (before imputation of missing values) of the 9829 identified proteins in the Iglesias-Gato dataset. In our dataset, we took the normalised ratios of the 3219 identified proteins. The heatmap below was created based on the pearson correlation values between the expression levels of 2673 proteins identified in both experiments. We can observe that cell lines clusterize apart from tissue data. This clustering is similar to the one obtained by in prostate cancer transcriptomics and phosphoproteomics (Drake et al. 2016) as well as for other cancer types and omics data (Domcke et al. 2013). It has also been observed for breast cancer that among different types of omics, proteins levels had the lowest correlation between tumors and cell lines (Jiang et al. 2016). Overall, these observations highlight the large dissimilarities between tissue samples and cell lines, emphasizing the need to obtain molecular profiles from both types of resources.

Domcke, S., Sinha, R., Levine, D. A., Sander, C. & Schultz, N. Evaluating cell lines as tumour models by comparison of genomic profiles. Nature Communications 4, 2126 (2013).

Drake, J. M. et al. Phosphoproteome Integration Reveals Patient-Specific Networks in Prostate Cancer. Cell 166, 1041–1054 (2016).

Jiang G, Zhang S, Yazdanparast A, et al. Comprehensive comparison of molecular portraits between cell lines and tumors in breast cancer. BMC Genomics. 2016;17 Suppl 7(Suppl 7):525.

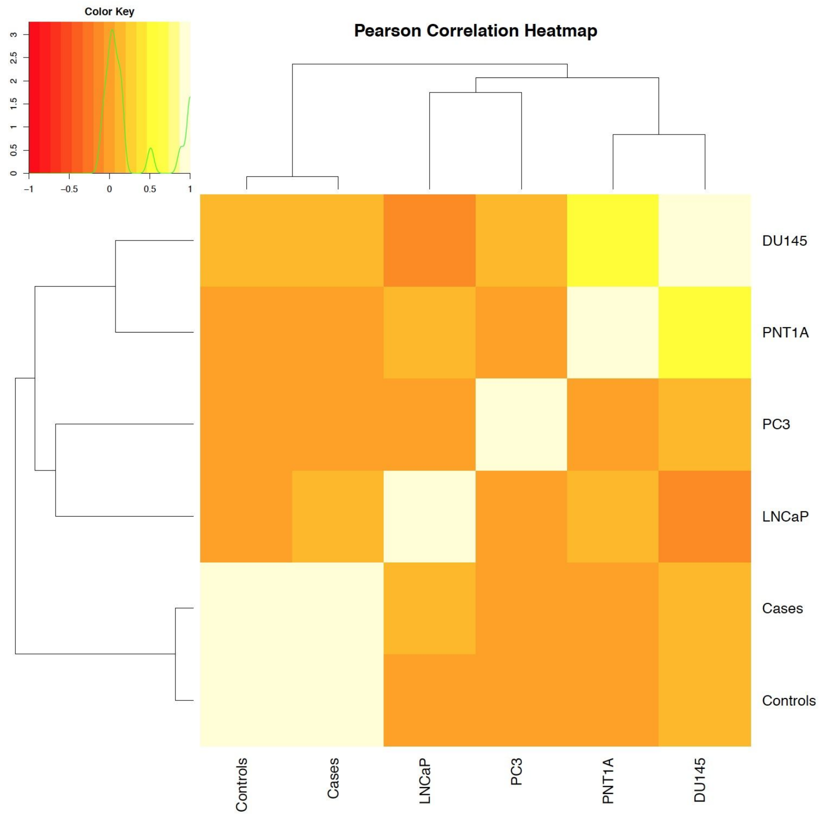

We also conducted a meta-analysis using the R package “MetaDE”. Our goal was to compare the protein level trends among tissue and cell lines. We used a Random Effects Model (REM) approach for the meta-analysis, since it allows heterogeneity in the effect sizes between the different datasets (Tissue VS cell lines). We took PNT1A as the reference for our SILAC experiment. Proteins were selected as those displaying an FDR corrected p-value < 0.05. The results are the following:

- **LNCaP**: 114 proteins have a similar behaviour in cell lines as compared to tumor tissues. This means that the expression level changes of these proteins between

LNCaP and PNT1A are similar to those between control tissue and tumorigenic tissue.

- **DU145**: 81 proteins have a similar behaviour.
- **PC3**: 73 proteins have a similar behaviour.

These results indicate that LNCaP is the cell line with protein levels displaying the most similar behavior as compared to the tumor tissue.

- Iglesia-gato 2018

We finally applied our previously mentioned comparison protocol to the data produced by Iglesias-Gato in 2018. In this case, they identify 9828 proteins, 2673 of them also match with our identified proteins. Here, we also observe that our protein expression data clusterize separately from the one resulting from tissue samples.

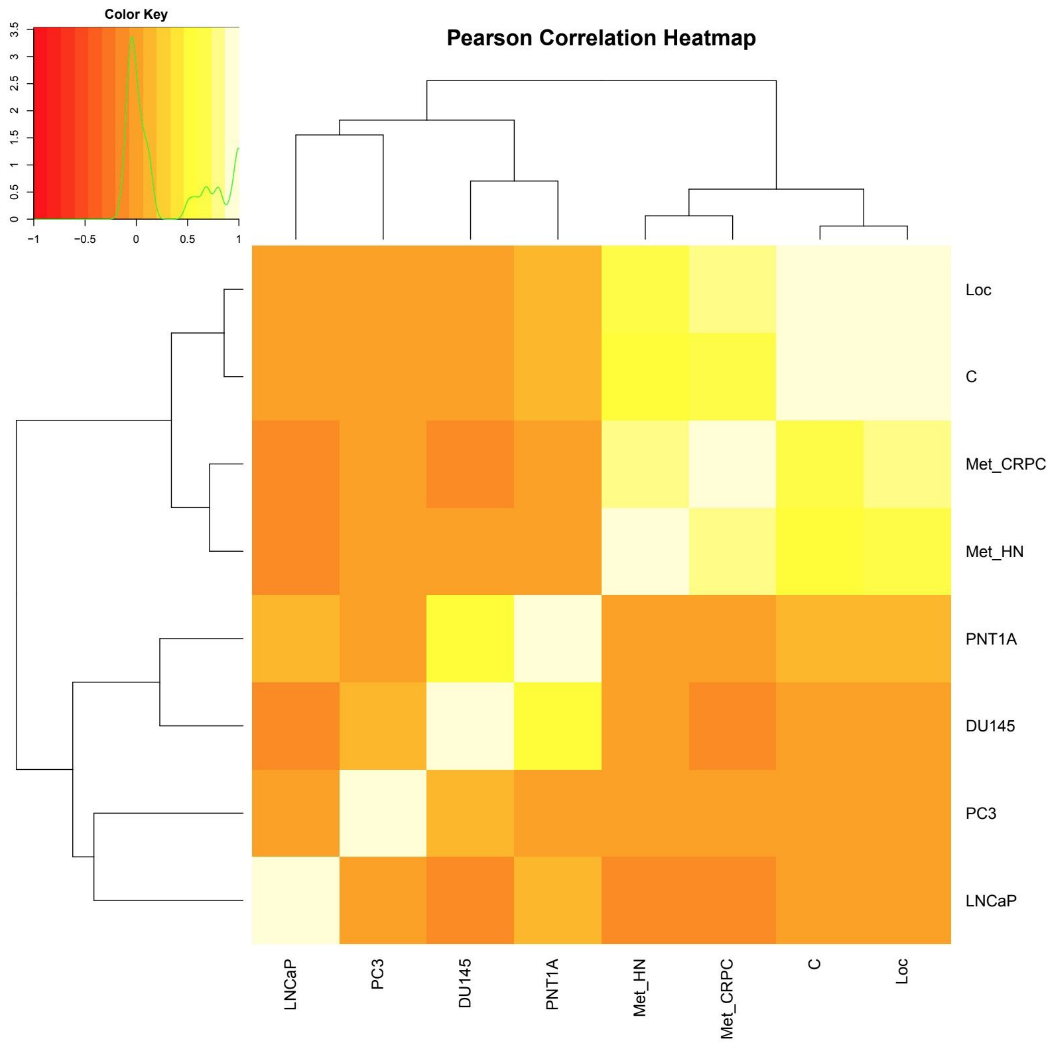

C: benign neighboring prostate tissue

Loc: gland localized prostate tumors (Primary tumors)
Met-HN: Bone metastasis Hormone-naive.
Met-CRPC: Bone metastasis Castration resistant prostate cancer

Overall, the main strengths and novelty of our work are to profile four widely used prostate cell lines and to do this both at the proteomics and phosphoproteomics levels. As shown in the previous comparisons, our datasets are complementary to the recent literature on prostate cancer transcriptomics, proteomics or phosphoproteomics, and could be useful for the community working with these cell lines in routine. Furthermore, we also propose a bioinformatics exploration of our datasets, as well as their integration into a molecular network, allowing the identification of processes that are not detectable with a traditional analyses of differentially expressed proteins/sites.

2) Please show if the data can be used for miRNA target prediction? It would be a good addition to the study as miRNA's have great potential as biomarkers in cancer.

This is indeed a very interesting point. During our review of the literature, we explored some interesting cases of miRNA-target interactions that could be relevant in the context of our prostate proteomics study (Vanacore et al. 2017). For instance, the regulation of VDAC1 by miR-29 is very appealing. VDAC1 is a mitochondrial protein directly involved in apoptosis (it promotes the apoptosis of tumor cells), and it harbours a miR-29a target site. The overexpression of miR-29a has been shown to result in downregulation of VDAC1 (Bargaje et al. 2012). The protein level profile of VDAC1 in the different prostate cells lines is shown below. Low levels in the expression of VDAC1 could lead to a decrease in the apoptosis of tumor cells resulting in more aggressive cancers as represented by PC3 and DU145 lines. miR-29 might be involved in this process, it would be interesting to profile its expression in these cell lines. We however acknowledge that we did not design our study to consider miRNA-target predictions. For instance, the network we are building considers proteins and phosphosites measured in our study, but we did not measured miRNA. It would be an attractive follow-up.

Vanacore D et al. Micrornas in prostate cancer: an overview. Oncotarget. 2017 Jul 25;8(30):50240-50251.

Bargaje R et al. Identification of novel targets for miR-29a using miRNA proteomics. PLoS One. 2012;7(8):e43243. doi:10.1371/journal.pone.0043243

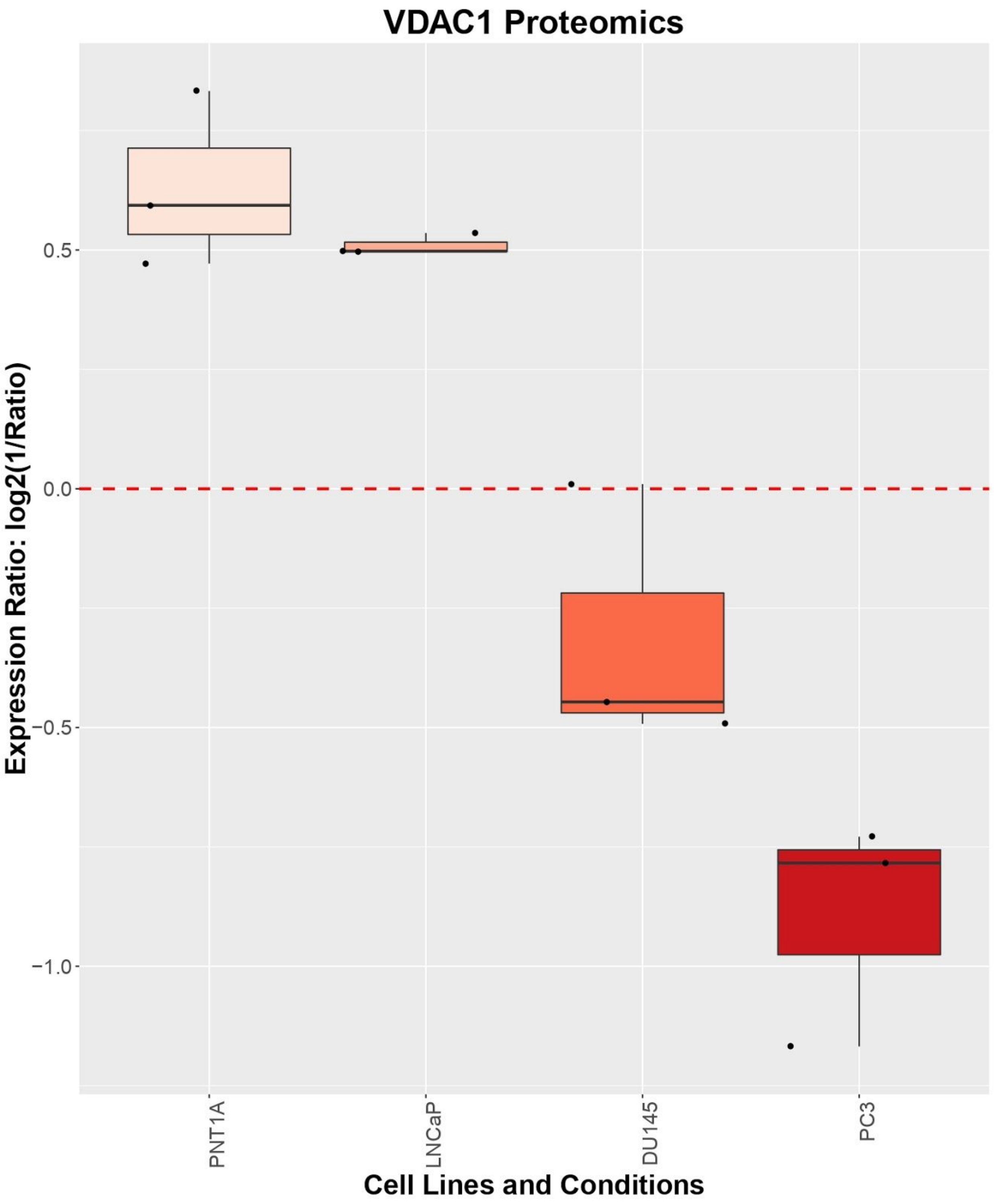

VDAC1 Proteomics
Expression Ratio: log2(1/Ratio)
0.5
0.0
−0.5
−1.0
PNT1A
LNCaP
DU145
PC3
Cell Lines and Conditions

3) Add PCA plot to show that each cell line has a unique protein and phosphor-protein signature and add Venn-diagrams to show common and unique expressors for a) cancer cell lines compared to non-tumorigenic cells b) castrate resistant cells compared to castrate sensitive cells.

We show below the PCA plots proteomics and phosphoproteomics data.

Proteomics:

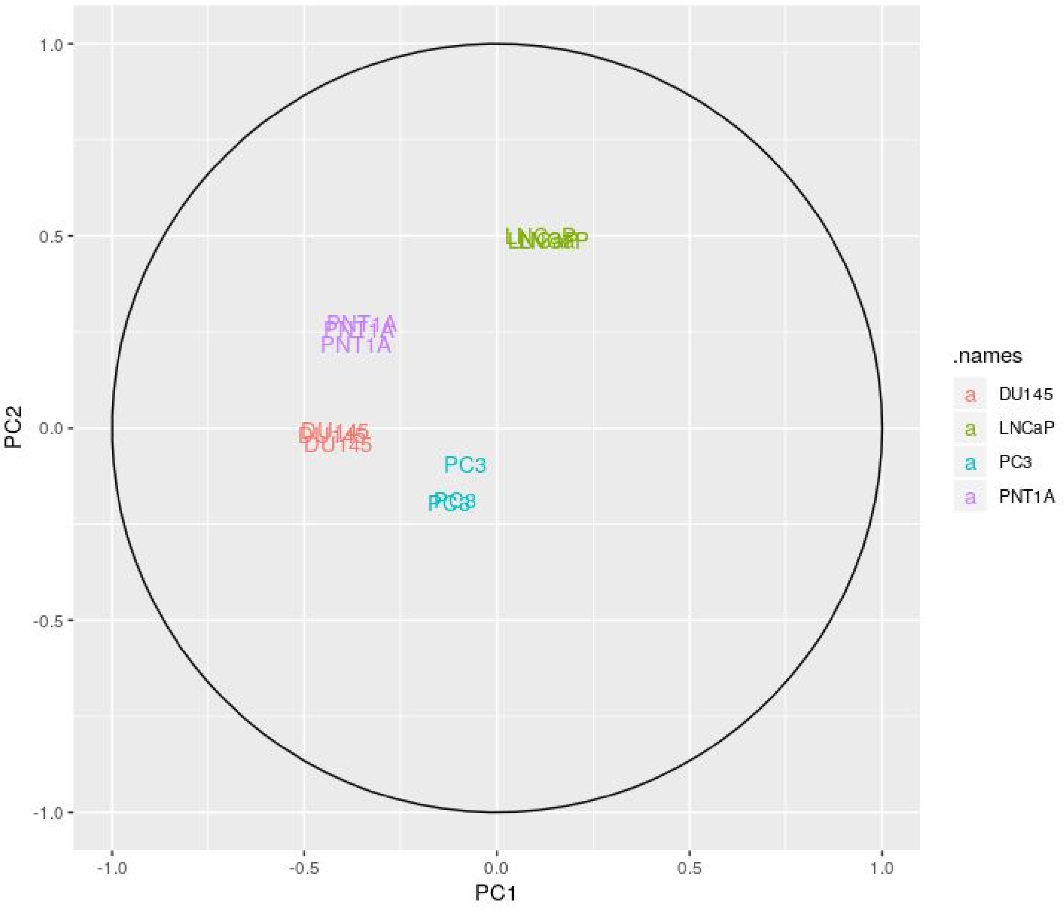

Phosphoproteomic: (please note that only two samples for the DU145 are considered, as commented in the manuscript)

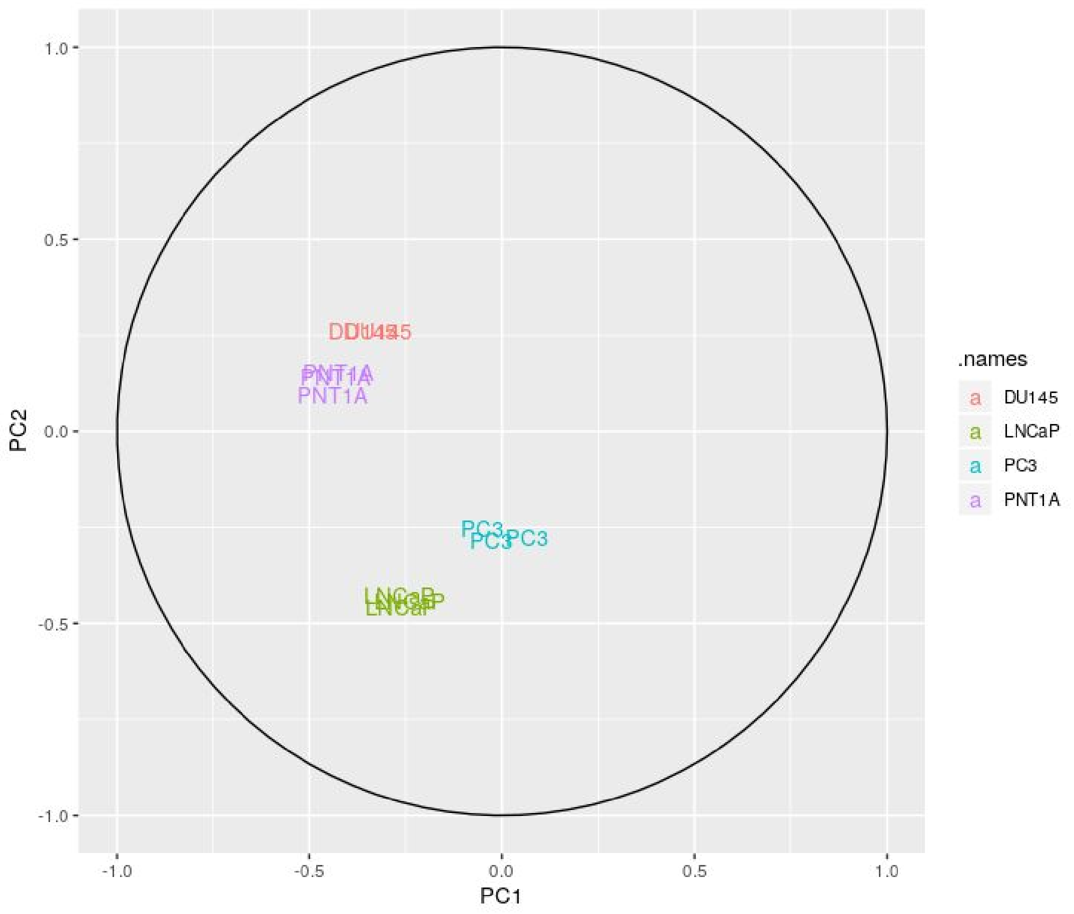

The PCA plots show in particular that the replicates cluster together. We decided not to include these figures in the manuscript because the main message would have been redundant with the clustering observed for the samples in the heatmaps already provided in the manuscript (Figure 1 D and E).

We next draw the Venn-diagrams to show proteins over or under expressed in a unique or in multiple cell lines.

a) Cancer cell lines (LNCaP, DU145 and PC3) compared to non-tumorigenic cells (PNT1A). As described in the manuscript, a t-test was applied to compare the expression value in the three cancer cell lines and the reference non-tumorigenic PNT1A cell line. FDR was used for multiple testing corrections, and the threshold of significance set to 0.1. The Venn diagrams are drawn from the datasets identified with this threshold.

Proteins overexpressed in the cancer cell lines when compared to PNT1A. These are the 13 proteins overexpressed in prostate cancer cell lines as compared to the non-tumorigenic cell line: ANXA11, CPNE3, CSTB, CTNNB1, DPP3, KRT18, NES, NTPCR, RAB5B, RAB7A, RPL36A, SRI and UBE2N;UBE2NL. Some of these proteins are detailed in the manuscript.

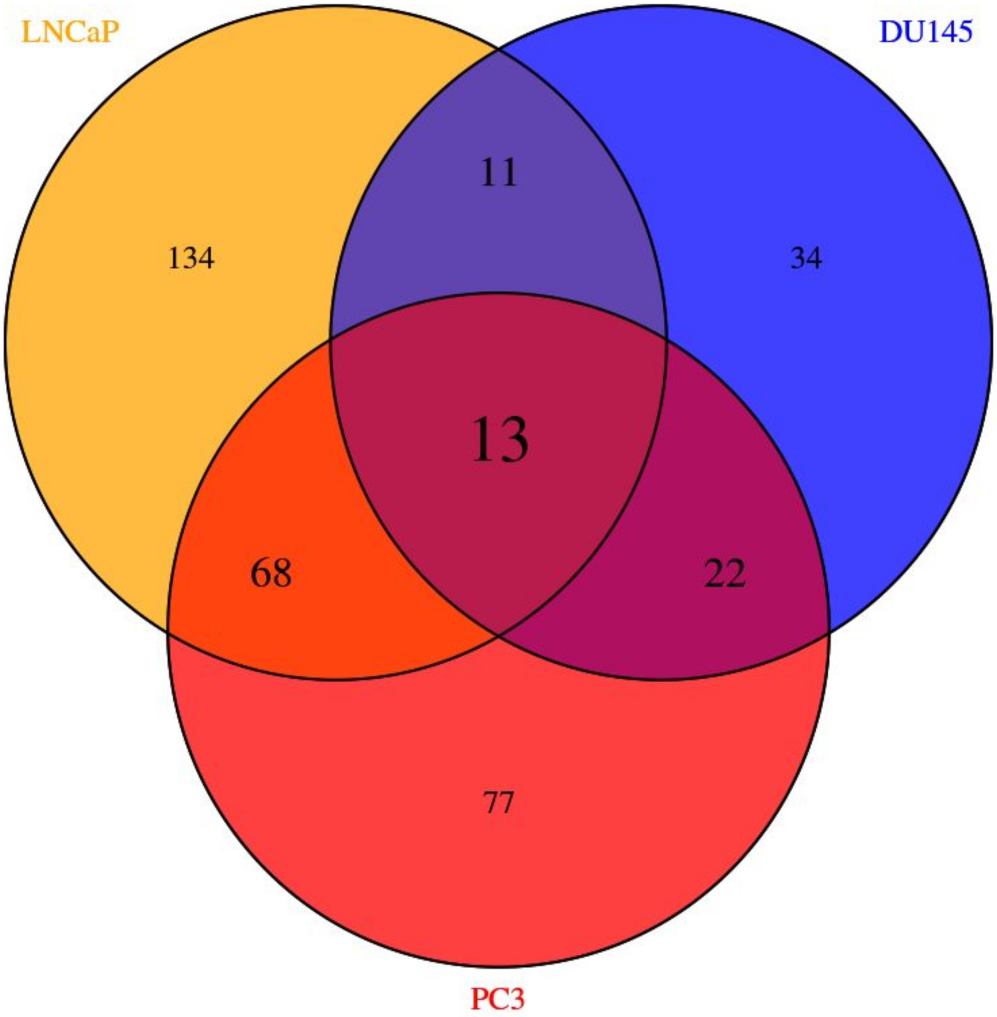

Proteins underexpressed in the cancer cell lines when compared to PNT1A. These are the 19 proteins commonly underexpressed in prostate cancer cell lines as compared to the non-tumorigenic cell line: CANX, CCT3, CCT5, CCT6A, CCT8, CTPS, DDX1, FLNC, GCLM, HNRNPA2B1, KARS, MYH10, NUDCD2, OCIAD1, PRDX1, RUVBL1, RUVBL2, SSRP1, TCP1. Here also, some of these proteins are mentioned in the manuscript.

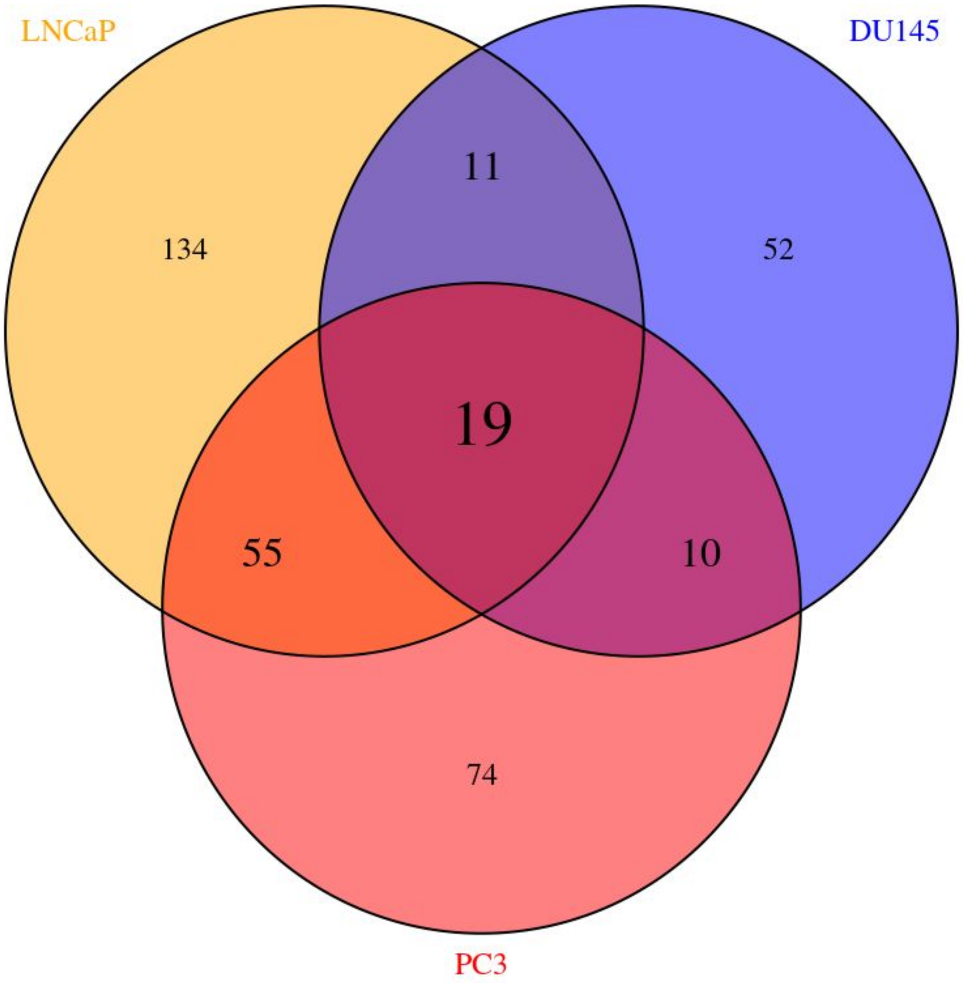

b) Castration resistant cell lines (DU145, PC3) compared to castration sensitive cell line (LNCaP). Pairwise comparisons of protein expression values between the castration-sensitive and the castration-resistant cell lines were performed with a t-test, and the threshold of significance set to 0.1 after FDR multiple testing corrections. The Venn diagrams are drawn from the datasets identified with this threshold.

Proteins overexpressed in castration-resistant cancer cell lines when compared to the LNCaP castration-sensitive cell line: The overexpressed proteins common to both resistant cell lines are present in the network displayed in the manuscript figure 4.

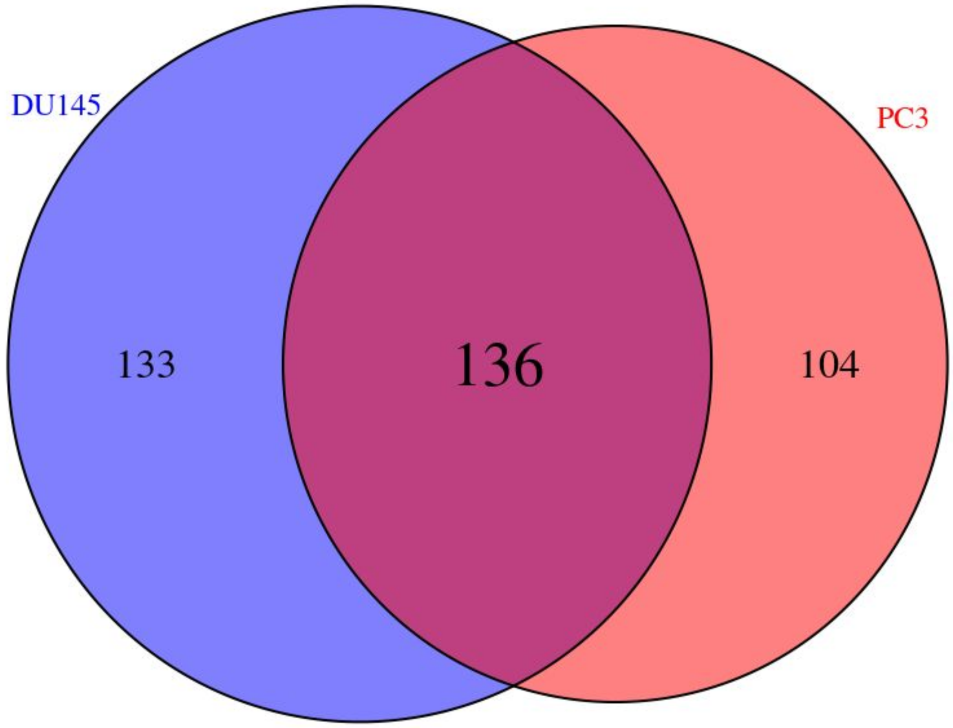

Proteins underexpressed in castration-resistant cancer cell lines when compared to the LNCaP castration-sensitive cell line: The proteins underexpressed in bother cell lines are present in the network displayed in the manuscript figure 4.

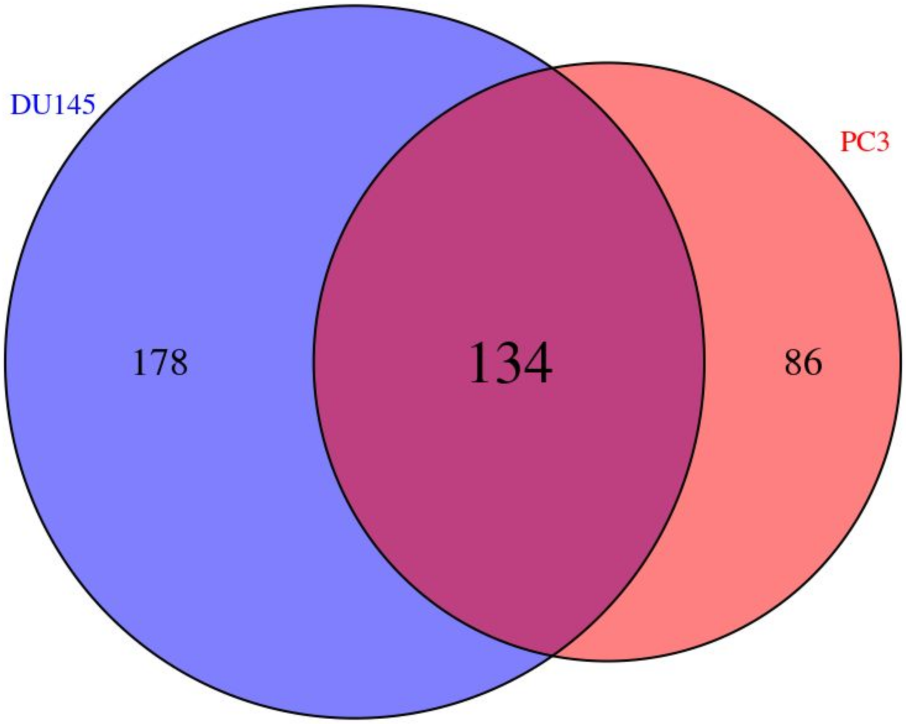

Overall, all the information necessary to draw Venn diagrams is available from the supplementary tables of proteomics and phosphoproteomics datasets. We think that these tables are more comprehensives and useful, because, contrary to the Venn diagram presented above, interested readers can select their own p-value threshold to consider a protein as over or under-expressed in a cell line. As the peer-review history might be published together with the paper, these Venn diagrams, as well as the PCA plots, would be nevertheless available to the interested readers.

4) Are there common expressors and unique expressors between DU145 and PC3. This could perhaps help identify candidate biomarkers and targets for highly metastatic and aggressive disease.

This is a very interesting comparison. We performed t-test comparisons between the two castration-resistant cell lines to determine differentially expressed proteins and phosphosites. We finally did not presented this information because we first aimed at exploring the molecular events driving the global progression of prostate cancer to resistance, i.e., we focused on the mechanisms that could be common in the two resistant cell lines as compared to the sensitive one. However, global similarities and differences between PC3 and DU145 can be appraised in the context of their comparison to PNT1A and LNCaP in the manuscript.

5) Show box-plots for expression levels of TAGLN2 and HNRNPN1 in an additional figure. Validation of differences in protein levels must be shown.

We assumed that the reviewer refers to TAGLN2 and HNRNPA1. These two proteins are interesting candidates because they have been associated with phosphosites significantly different from one cell line to another according to an ANOVA test. We are not sure to understand the comment on the validation of differences in protein levels. Both proteins are housekeeping (top 10% of most expressed proteins in all cell lines according to iBAQ values). All proteins discussed in the manuscript have been identified in the proteomics experiment with an FDR < 1%. TAGLN2 is in addition under-expressed in LNCaP cell line (FDR=0.007), and over-expressed in DU145 (FDR=0.02) and PC3 (FDR=0.049) cell lines, as compared with PNT1A, and over-expressed in resistant compared to sensitive cell lines (FDR=0.01). As suggested by the reviewer, box-plots describing the protein and phosphosite levels of TAGLN2 and HNRNPA1 have been generated and are included in the submission of the new version of the manuscript as supplementary figures 4 and 5.

5) The resolution is images in the figures needs to be increased. Figures are hard to read as of now.

We increased the resolution the final version of the manuscript. In addition, we increased the size and quality of the labels in figures 2 and 3.

Reviewer #3:

The manuscript from Katsogiannou et al. showed a large SILAC-based Mass Spectrometry experiment that allowed to map the proteomes and phosphoproteomes of PNT1A, LNCaP, DU145 and PC3 prostate cancer cell lines, and reveal different signaling networks associated with the cellular context of each cell line, possibly

reflecting the pathological features of human Prostate Cancer (hormonal status, ability to metastatize etc.).
The experimental data are strong, rigorous and well presented, in particular the deep comparison of the four cell lines for the identification of the housekeeping proteome vs the most significant variations across the samples.

We thank the reviewer for his encouraging comments and for the critical points discussed, which help us to improve the quality of our manuscript.

However, some critical points emerged, and should be clarified/investigated more in depth:

1. The androgen-dependent/castration-sensitive cell line LNCaP has been compared with the androgen-independent/castration-resistant cells DU145 and PC3. However, the best in vitro models for comparing these two PrCa conditions would be LNCaP vs C4-2. Why the authors did exclude C4-2 cells from their high-throughput analysis?

Several other PCa models exist, such as C4-2, a particularly interesting LNCaP-derived cell line that constitutes a compelling androgen-independent model. We however had to make a choice in the number of cell lines during the initial design of the study, because of the amount of experimental material and subsequent data produced. We decided not to work with derived cell lines (from LNCaP or other cells), because of the genetic proximity. We wanted instead to identify mechanisms of resistance that could have emerged in genetically different cell lines, derived from separate metastasis. The cells lines described in our study are four of the most widely used cell lines in prostate cancer research (Pienta et al. 2008, Cunningham et al. 2015) that we routinely use in the lab.

Pienta KJ, Abate-Shen C, Agus DB, Attar RM, Chung LW, Greenberg NM, Hahn WC, Isaacs JT, Navone NM, Peehl DM, Simons JW, Solit DB, Soule HR, VanDyke TA, Weber MJ, Wu L, Vessella RL. The current state of preclinical **prostate** cancer animal models. **Prostate**. **2008**May 1;68(6):629-39. doi: 10.1002/pros.20726

CunninghamD, You Z. In vitro and in vivo model systems used in **prostate** cancer research. J Biol Methods. **2015**;2(1). pii: e17.

2. The ANOVA analysis of the proteomics/phosphoproteomics data highlighted several proteins/phosphosites that vary significantly in various comparisons (benign vs malignant, CR vs CS etc.). For some of these variations, the authors even claimed that “these proteins could constitute markers of oncogenic transformation”. To support this kind of statements, the authors should provide “wet-lab” validations of their

high-throughput results, at least on representative targets among those described in the text (e.g. Septin-9, TAGLN2, HNRNPA1, RAB5B/RAB7A, TriC/CCT complex, TP53BP1 pSer-500 and pThr-1056, DDX10 pSer-539 etc). This type of validation would also help the authors focusing on the most important pathways, rather than leaving the reader with a comprehensive description of all signaling networks potentially involved in the regulation of PrCa malignant progression.

The generation of the SILAC proteomics and phosphoproteomics dataset from the 4 cell lines and with 3 replicates was a large and complex experimental work, and we decided to focus our subsequent analyses on the in-depth data exploration of this dataset rather than on functional experimental validation of cherry-picked candidates. In the design of our study, we took inspiration from references in the proteomics field, in particular from Matthias Mann lab papers on cell line proteomics characterizations (Lundberg et al. 2010, Geiger et al. 2012), but also from a reference in prostate cancer analysis in which the authors produced phosphoproteomics and genomics for prostate cancer, both on cell lines and real tumor tissue (Drake et al. 2016). Similarly to these studies, we concentrated our efforts on the data generation and their exploration, with the objective to produce a resource that could subsequently be used as a starting point for further hypothesis and experimental investigations (by us and others, as, importantly, we make all data available [ProteomeXchange Consortium (www.proteomexchange.org) via the PRIDE partner repository with datasets identifiers PXD004970 and PXD004992.]).

Lundberg, E. et al. Defining the transcriptome and proteome in three functionally different human cell lines. Molecular Systems Biology 6, (2010).

Geiger, T., Wehner, A., Schaab, C., Cox, J. & Mann, M. Comparative Proteomic Analysis of Eleven Common Cell Lines Reveals Ubiquitous but Varying Expression of Most Proteins. Mol Cell Proteomics 11, (2012).

Drake, J. M. et al. Phosphoproteome Integration Reveals Patient-Specific Networks in Prostate Cancer. Cell 166, 1041–1054 (2016).

Minor points:

1. Page 8, Lanes 303-305): DU145 are derived from CNS metastasis, and PC3 from bone metastasis. Please revise the sentence.

Thanks for catching this mistake! This has been corrected.

2. Figure 2: it should be useful to include a “title/legend” to each bar graph (e.g. LNCaP vs PNT1A in the panel A), showing the comparisons as described in the figure legend text.

Done

3. Figure 3: similar to the previous point, it should be useful to include a “visible” title/legend to each panel (e.g. DU145/PC3 vs LNCaP in the panel A, and CR vs CS on top of the panel B).

Done. We have now included a title for each subfigure to further clarify the different plots as suggested. Moreover, we increased the size of the label titles and numbers in the axis. Done

6. PLOS authors have the option to publish the peer review history of their article (what does this mean?). If published, this will include your full peer review and any attached files.

If you choose “no”, your identity will remain anonymous but your review may still be made public.

Do you want your identity to be public for this peer review? For information about this choice, including consent withdrawal, please see our Privacy Policy.

Reviewer #1: No

Reviewer #2: No

Reviewer #3: Yes: Marco Trerotola, PhD
Laboratory of Cancer Pathology
Center for Advanced Studies and Technology (CAST)
Department of Medical, Oral and Biotechnological Sciences
"G. d'Annunzio" University of Chieti-Pescara (Italy)

[NOTE: If reviewer comments were submitted as an attachment file, they will be attached to this email and accessible via the submission site. Please log into your account, locate the manuscript record, and check for the action link "View Attachments". If this link does not appear, there are no attachment files to be viewed.]

While revising your submission, please upload your figure files to the Preflight Analysis and Conversion Engine (PACE) digital diagnostic tool, https://pacev2.apexcovantage.com/. PACE helps ensure that figures meet PLOS requirements. To use PACE, you must first register as a user. Registration is free. Then, login and navigate to the UPLOAD tab, where you will find detailed instructions on how to use the tool. If you encounter any issues or have any questions when using PACE, please email us at figures@plos.org. Please note that Supporting Information files do not need this step.
